# Differences in spatial cognitive performance and cognitive load between pilots and student pilots in virtual reality environments

**DOI:** 10.3389/fpsyg.2026.1903114

**Published:** 2026-08-12

**Authors:** Jingyun Lu, Jie Wang, Chao Wang, Wenjia He, Jing Sun, Lihong Zhai, Shengguang Yan, Zhanguo Jin

**Affiliations:** 1 School of Public Health, North China University of Science and Technology, Tangshan, China; 2 Second Affiliated Hospital of Anhui Medical University, Hefei, Anhui, China; 3 The Department of Vertigo Medical Centre, Air Force Medical Center.PLA, Beijing, China

**Keywords:** aviation psychology, cognitive load, maze navigation, mental rotation, pilots, spatial cognition, student pilots, virtual reality

## Abstract

**Purpose:**

The factors underlying the differences in spatial cognition between pilots and flight trainees remain unclear; therefore, this study conducts an exploratory investigation into these differences and their relationship with cognitive load. This study compared experienced pilots and student pilots on two spatial cognitive tasks, examined associations among pilot status, task performance, and subjective cognitive load, and examined whether subjective cognitive load served as a statistical mediator of the association between pilot status and spatial performance.

**Methods:**

Eighty pilots and 80 student pilots with no flight experience were enrolled. Participants completed a 6 × 6 virtual-reality maze navigation task and an R-letter mental rotation task. Subjective cognitive load was assessed using an adapted NASA-TLX questionnaire.

**Results:**

Significant group differences were observed in task performance and subjective cognitive load. Higher subjective cognitive load was associated with longer maze completion time and lower mental-rotation accuracy. Regression analyses identified subjective cognitive load as the strongest statistical predictor of maze completion time and R-letter accuracy. Cross-sectional mediation analysis showed a significant indirect association between pilot status and maze completion time through subjective cognitive load. In the pilot subgroup, flight experience did not show a significant association with maze performance.

**Conclusion:**

Experienced pilots showed better performance on selected spatial cognitive indicators compared with student pilots, particularly during VR maze navigation. These group differences were statistically associated with lower subjective cognitive load in the experienced pilot group; however, causal inferences are limited by the cross-sectional design.

## 1 Introduction

Spatial cognition refers to the mental representation and processing of object location, orientation, directional awareness, and spatial relationships (Langlois et al., 2024). As a core cognitive function for pilots in navigation, instrument interpretation, and emergency response, spatial cognition involves rapid comprehension, dynamic updating, and precise manipulation of three-dimensional spatial relationships, thereby affecting flight safety and mission effectiveness (Burles and Iaria, 2023).

The neural basis of spatial cognition depends on coordinated activity across multiple brain systems rather than isolated activity within a single region. Important components include grid cells in the medial entorhinal cortex, spatial mapping regions in the parietal cortex, and vestibular-visual integration pathways. Grid cells provide a coordinate framework for spatial representation, whereas the parietal cortex integrates visual, proprioceptive, and vestibular signals to form spatial reference frames (Bellmund et al., 2018; Zhang et al., 2023).

Spatial cognition is a critical competency for pilots, especially during navigation, attitude awareness, and emergency response. Aviation accidents are closely linked to human factors, with spatial disorientation, navigation errors, and inadequate adaptation to dynamic environments being particularly important operational risks (Song and Cui, 2017). Previous work suggests that pilot performance is related to flight experience and executive functioning (Causse et al., 2011), but the ways in which pilot status and experience are associated with different spatial task types remain insufficiently understood.

Mental rotation and spatial navigation tasks may involve partially distinct cognitive operations. Maze navigation depends strongly on environmental cues, route integration, and spatial orientation, whereas R-letter mental rotation relies more on the analysis and transformation of abstract spatial relationships (Mallot and Basten, 2009; Shepard and Metzler, 1971). Cognitive load theory (Sweller et al., 1998) provides a framework for understanding these differences. CLT posits that instructional design and expertise shape the allocation of limited working memory resources. Specifically, the expertise reversal effect (Burles and Iaria, 2023) suggests that instructional supports beneficial for novices may become redundant or even detrimental for experts whose schemas are well-automated. In aviation contexts, extensive training and flight experience have been proposed to be associated with more efficient processing of spatial information, which may influence perceived cognitive demands during navigation tasks. Accordingly, we hypothesized that experienced pilots would report lower subjective cognitive load during spatially demanding VR tasks compared with student pilots.

Virtual reality (VR) provides an ecologically enriched analogue for spatial cognition assessment. Compared with traditional two-dimensional paper-and-pencil or computer-screen tests, VR environments allow dynamic scene control, embodied interaction, and real-time feedback (Costa et al., 2018). However, it is crucial to acknowledge the limitations regarding ecological validity. The VR maze is an analogue task; it does not replicate the complex psychomotor demands, G-force effects, or life-critical decision-making pressures inherent in actual flight. Furthermore, the R-letter mental rotation task, while tapping into spatial visualization, is somewhat distal from operational aviation demands such as interpreting attitude indicators or managing airspace geometry. The high accuracy rates observed in both groups also suggest a potential ceiling effect, limiting the sensitivity of the R-task to discriminate expert performance. Therefore, implications for operational aviation training should be considered preliminary.

This study aimed to investigate whether pilots and student pilots differ in VR maze navigation and R-letter mental rotation performance, whether pilots report lower subjective cognitive load, and whether subjective cognitive load statistically accounts for the association between pilot status and maze task performance. We hypothesized that: (1) experienced pilots would show better performance on selected VR-based spatial cognitive indicators compared with student pilots; (2) pilots would report lower subjective cognitive load; and (3) subjective cognitive load would show a significant indirect association between pilot status and maze task performance.

## 2 Methods

### 2.1 Participants

A total of 80 qualified pilots and 80 qualified student pilots were enrolled. No severe adverse events occurred during the study, and no participants withdrew. Pilots were healthy adult pilots. Student pilots were trainees at a flight academy and had no flight experience.

General inclusion criteria were as follows: age at least 18 years; uncorrected visual acuity of at least 1.0; right-handedness; no history of severe three-dimensional motion sickness or VR-related discomfort; no history of neuropsychiatric disease; no spatial orientation disorder or navigation difficulty; no recent acute infection or infection-related symptoms; no recent use of drugs affecting cognitive function; no alcohol or caffeine intake within 24 h before testing; no sleep deprivation within 48 h before testing; and provision of written informed consent.

Exclusion criteria were acute vestibular syndrome, benign paroxysmal positional vertigo, severe anxiety or claustrophobia, recent head trauma, marked VR-related discomfort, or acute illness associated with spatial disorientation or navigation difficulty. Because pilot status was naturally associated with differences in age and flight experience, these variables were considered important covariates during statistical analyses.

### 2.2 Testing tools and software

**Figure 1:**
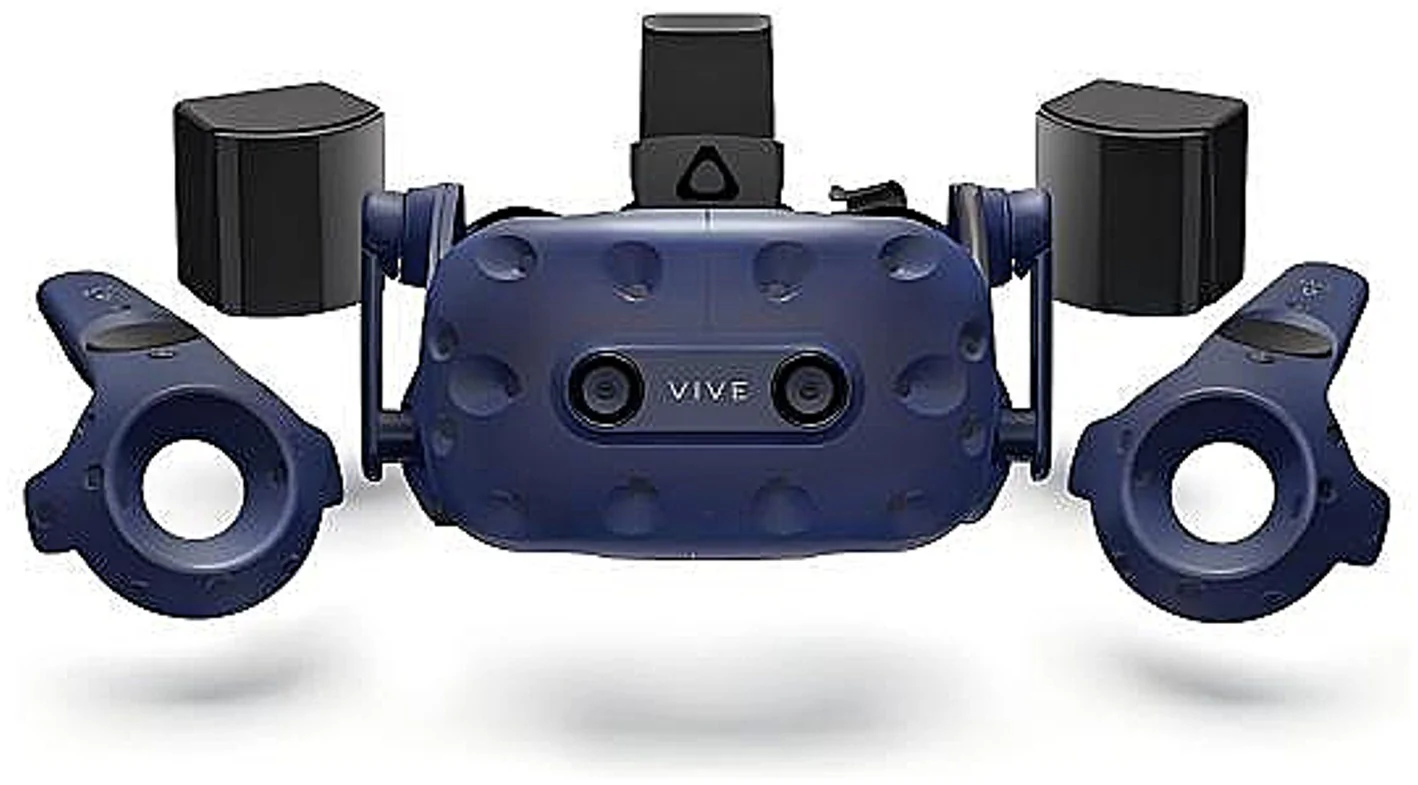
HTC VIVE controller, headset, and locator used in the VR experiment.

The VR system was developed using the Unity3D engine and SteamVR plugin framework and was implemented with the HTC VIVE VR headset, hand controllers, and base stations (Figure 1). The system used Unity3D physics functions to support spatial interaction and real-time recording of task performance through the controller.

### 2.3 Maze test and procedure

The test prototype is derived from the maze sub-test of the classic psychological assessment tool, the Wechsler Intelligence Scale for Children Revised (WISC-R) (Costa et al., 2018). Using 3D modeling technology, it was redeveloped into a virtual environment, including walls, floors and ceilings. The experiment has an observation stage: Participants have 30 s to view the global map before entering the maze, and their initial position is set at the entrance. If they lose their trajectory during navigation, pressing the trigger button on the back of the controller will activate the temporary navigation and display the real-time coordinates. Each level contains four maze layouts with different path configurations, but the effective path lengths are the same and the number of turns is balanced.

**Figure 2:**
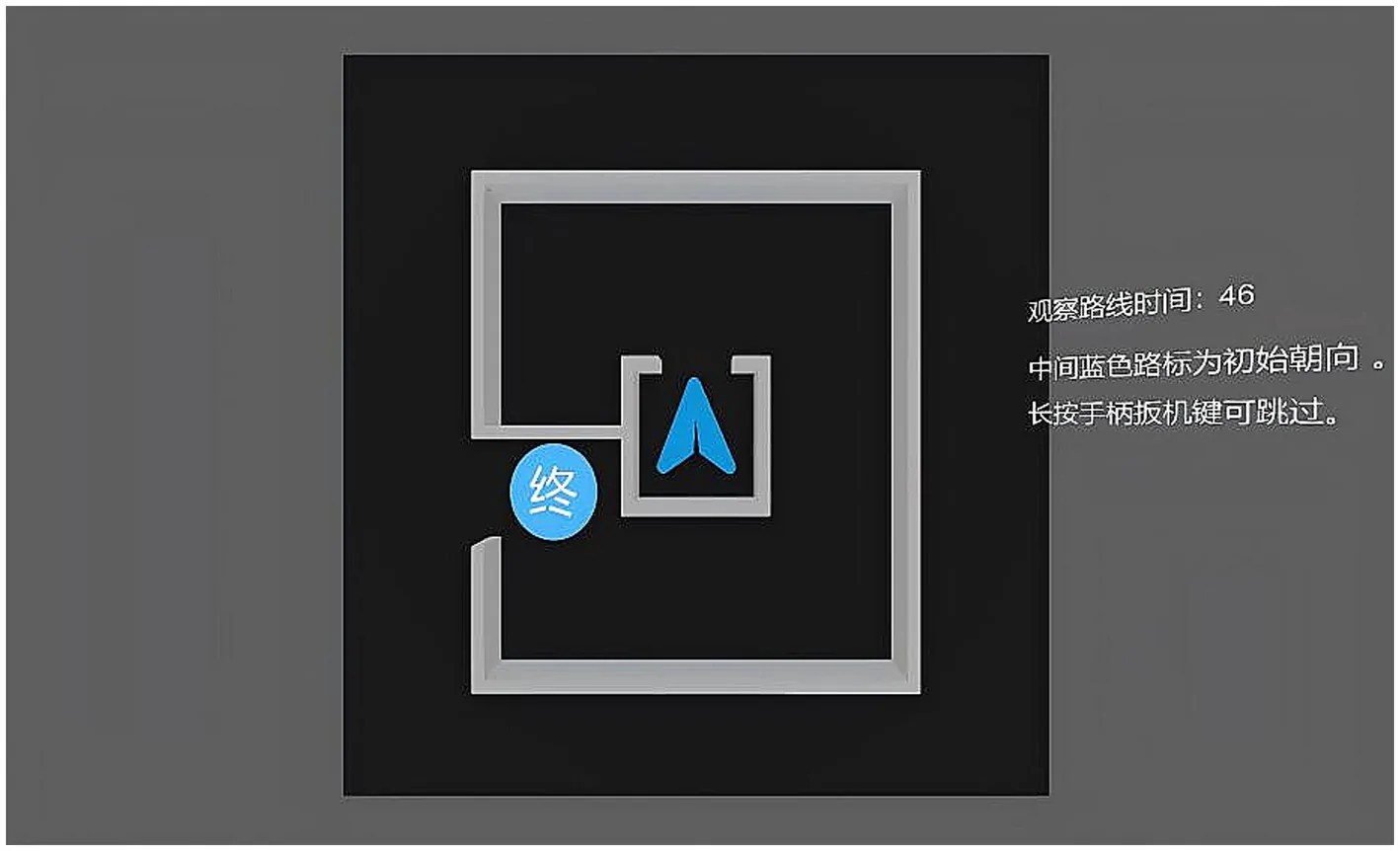
The 2 × 2 practice maze used for experimental guidance.

**Figure 3:**
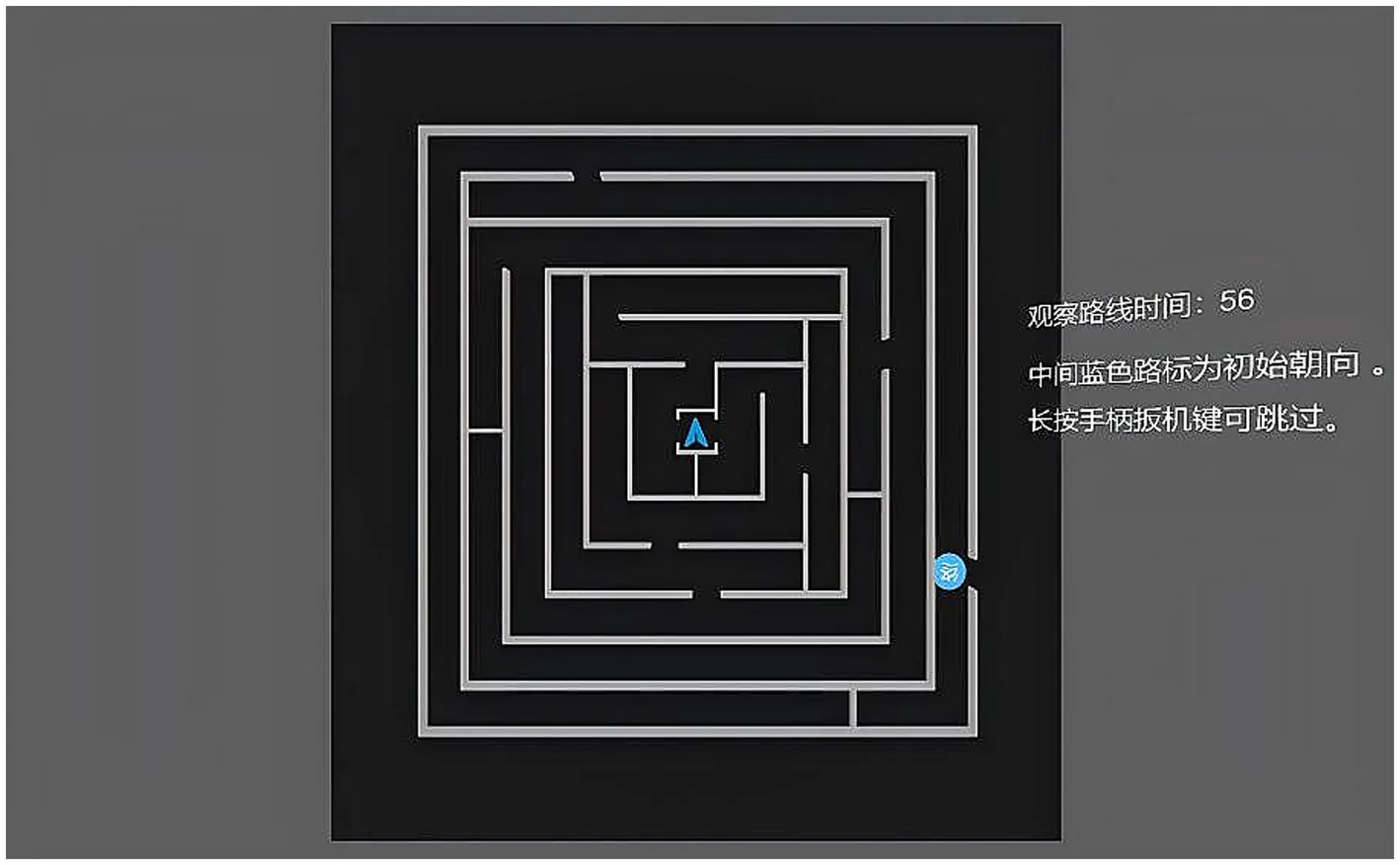
Example of one formal 6 × 6 maze map.

The evaluation method for training efficacy is as follows: Participants completed the 2 × 2 matrix maze task without external guidance (Figure 2); once they mastered the procedure, each participant was randomly assigned to one of five maps for the formal 6 × 6 maze test (Figure 3), with all maps having comparable effective path lengths and number of turns. For subsequent analysis, the average maze completion time and error count across the three tests were calculated (Zhang et al., 2023).

### 2.4 R-letter mental rotation test and procedure

**Figure 4:**
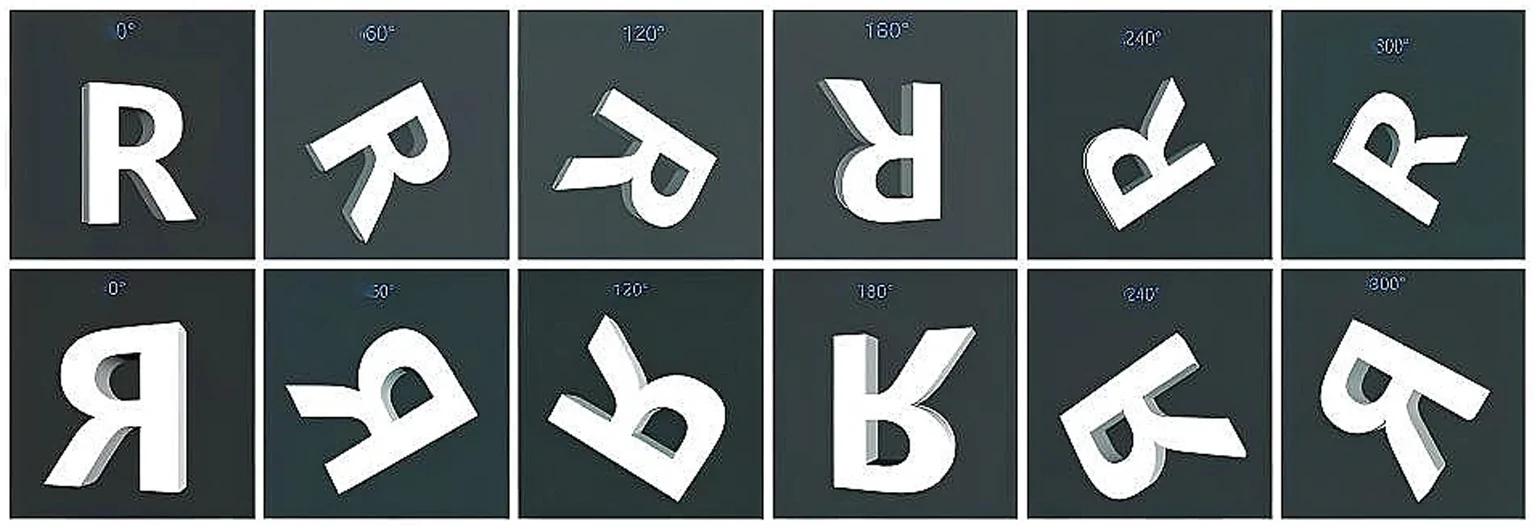
R-letter rotation angles. The first row shows normal stimuli, and the second row shows mirror-reversed stimuli.

This test is based on the classic mental rotation experimental paradigm developed by Shepard and Metzler (1971). Each type contains six rotation angles (0°, 60°, 120°, 180°, 240°, 300°). During the experiment, the system randomly presents a stimulus model each time. Participants were instructed to determine as quickly and accurately as possible whether each stimulus was a normal or mirror-reversed letter R, regardless of its rotation angle. The task comprised 12 trial sessions. Reaction time was defined as the interval from stimulus presentation to participant’s key response (Guo et al., 2025). Figure 4 shows all rotation angles of R. The first row represents forward rotation, while the second row shows mirror rotation.

### 2.5 Spatial cognitive load assessment

Immediately after completing the tasks, participants completed the adapted NASA-TLX-based subjective cognitive load questionnaire. The questionnaire included 18 items covering six dimensions: Mental Demand, Physical Demand, Temporal Demand, Performance, Effort, and Frustration, with three items for each dimension. Each item was rated on a 1–5 scale. The total score ranged from 18 to 90, with higher scores indicating higher subjective cognitive load (Hart and Staveland, 1988). Consistent with the original NASA-TLX protocol, raw scores for the Performance dimension were reverse-coded so that higher scores uniformly indicated higher workload. A global score was calculated by summing the six dimension scores (range 18–90). The adapted scale showed acceptable internal consistency in the present sample (Cronbach’s *α* = 0.825). Exploratory factor analysis indicated that the principal component explained 63.64% of the variance (KMO = 0.817, Bartlett test *p* < 0.01).

### 2.6 Statistical analysis

Data analysis was conducted using SPSS 24.0. First, descriptive statistics were compiled for demographic characteristics and task performance between the two groups. Continuous variables with normal distribution were expressed as mean ± standard deviation, while those with non-normal distribution were presented as median and percentage of the interquartile range. The Shapiro–Wilk test was performed to assess data normality; independent samples T-tests were applied to normally distributed data, whereas the Mann–Whitney U test was used for non-normal data. All statistical analyses were conducted as two-sided tests with a significance level of 0.05 subsequently.

Spearman correlation analysis was used to examine associations among flight experience, cognitive load, and task performance. Hierarchical multiple linear regression was then conducted to examine whether cognitive load predicted maze completion time and R-rotation accuracy after controlling for age, group, and flight experience. Exploratory Statistical Mediation Analysis were performed using Hayes’s PROCESS macro Model 4 with 5,000 bias-corrected bootstrap samples. Age was included as a covariate. The indirect effect was considered significant when the 95% bootstrap confidence interval did not include zero.

## 3 Results

### 3.1 Descriptive statistics

**Figure 5:**
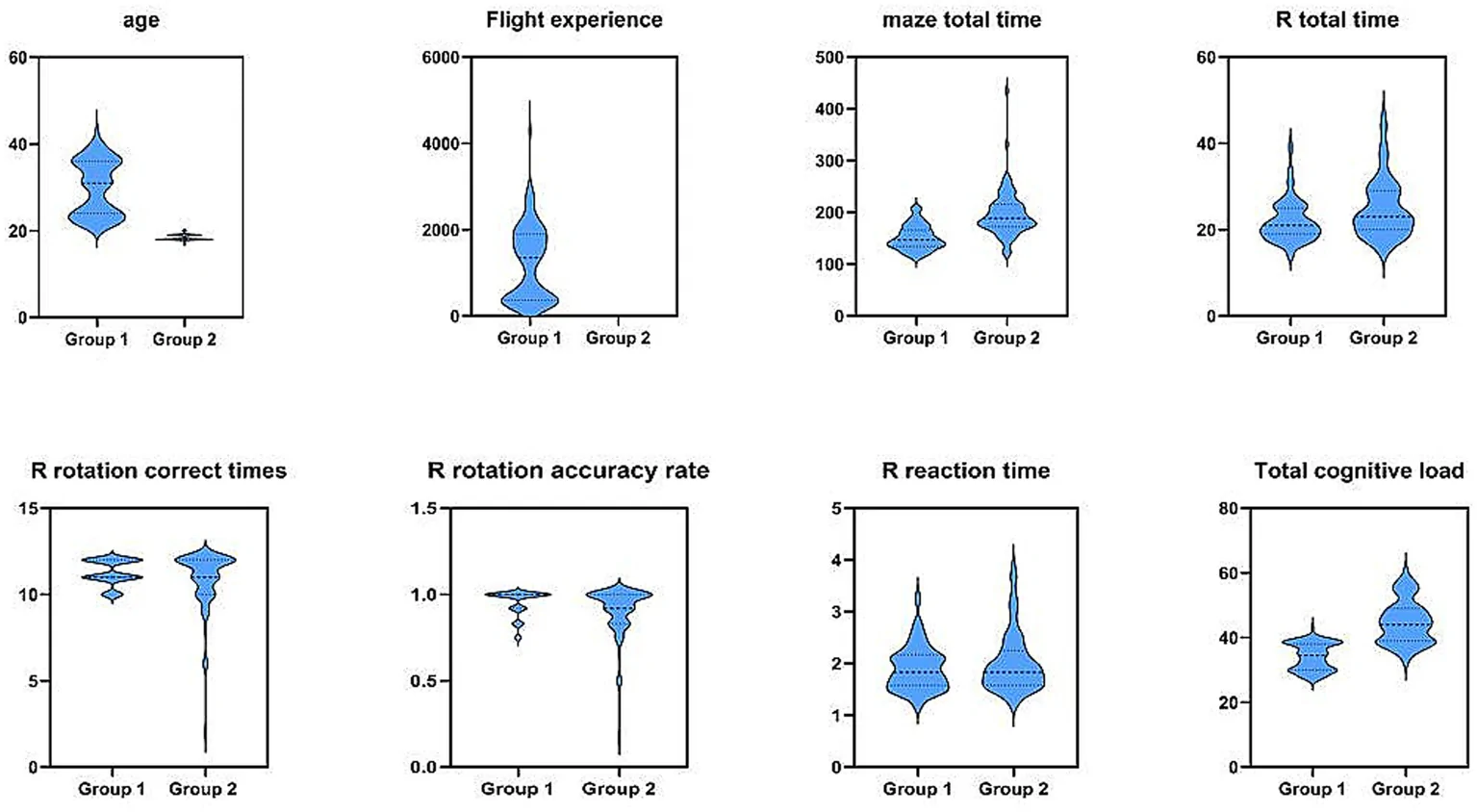
Violin plots comparing demographic indicators, spatial task performance, and cognitive load between groups.

This study compared pilots and student pilots in age, flight experience, maze error count, total maze time, R-letter total time, R-letter accuracy count, R-letter accuracy rate, R-letter reaction time, and total cognitive load. Pilots were older than student pilots and had substantially greater flight experience (Figure 5). Pilots completed the maze task in less time, whereas maze error count did not differ significantly between groups. In the R-letter rotation task, pilots showed shorter total processing time, more correct responses, and higher accuracy in unadjusted comparisons. Student pilots reported higher total cognitive load. Given the substantial and nearly non-overlapping age distribution between groups, which introduced severe confounding between group status and age, we employed a rank-based regression approach. To mitigate violations of parametric assumptions and reduce the influence of extreme values (Wood and Games, 1990) all continuous outcome variables (e.g., maze time, accuracy count) and continuous predictors (age, flight experience, cognitive load) were rank-transformed prior to model fitting. Consequently, the unstandardized (B) and standardized (beta) coefficients in Tables 1, 2 represent the change in rank of the dependent variable associated with a one-unit increase in the rank of the predictor. Variance Inflation Factors (VIFs) were inspected to assess multicollinearity; all VIFs were below 2.5, indicating acceptable levels. Effect sizes (R^2^) and 95% confidence intervals for regression coefficients are reported. While adjusting for age-related confounding without relying on the assumptions required for Analysis of Covariance (ANCOVA). Descriptive statistics are presented as medians (IQR) due to the skewed distribution of the raw data. After age adjustment, pilots still showed shorter total maze time, more correct R-letter responses, and lower cognitive load (Table 3).

**Table 1:** Linear regression model predicting maze completion time.

Predictor	Unstandardized B	SE	Beta	t	*p*
Constant	8.209	30.760	—	0.267	0.790
Age	0.418	1.250	0.072	0.335	0.738
Flight experience	−0.006	0.009	−0.123	−0.656	0.513
Group	0.282	8.297	0.003	0.034	0.973
Cognitive load	4.042	0.401	0.732	10.069	<0.001

B denotes the unstandardized regression coefficient, SE denotes standard error, and beta denotes the standardized regression coefficient. Group coding: 0 = pilot group, 1 = student pilot group.

**Table 2:** Linear regression model predicting R-letter accuracy count.

Predictor	Unstandardized B	SE	Beta	t	*p*
Constant	13.767	1.455	—	9.462	<0.001
Age	−0.018	0.059	−0.096	−0.304	0.762
Flight experience	9.252 × 10^-5	0.000	0.059	0.215	0.830
Group	−0.126	0.392	0.046	−0.320	0.749
Cognitive load	−0.055	0.019	−0.308	−2.885	0.004

**Table 3:** Demographic characteristics and task performance of the two groups.

Variable	Pilot group (*n* = 80)	Student pilot group (*n* = 80)	*Z*	*p*	Age-adjusted statistic	Age-adjusted *p*
Age (years)	31 (24, 36)	18 (18, 19)	−11.097	<0.001	—	—
Flight experience (hours)	1,350 (365, 1900)	0	−11.677	<0.001	—	—
Maze error count	1 (0, 1)	1 (0, 2)	−1.818	0.069	−0.340	0.734
Maze total time (s)	147 (134, 166)	189 (172, 216)	−7.639	<0.001	−2.392	0.017
R total time (s)	22 (19, 25)	23 (20, 29)	−2.758	0.006	−1.512	0.131
R accuracy count	12 (11, 12)	11 (10, 12)	−2.380	0.017	−2.083	0.037
R rotation accuracy rate	1.00 (0.92, 1.00)	0.92 (0.83, 1.00)	−2.489	0.013	−1.464	0.143
R rotation time (s)	1.75 (1.58, 2.08)	1.92 (1.67, 2.42)	−2.758	0.006	−1.512	0.131
Total cognitive load	35 (30, 38)	44 (39, 49)	−8.806	<0.001	−2.471	0.013

Group was coded as 0 = pilot and 1 = student pilot. *p* values below 0.001 are reported as <0.001. Values are presented as median (interquartile range). Unadjusted between-group comparisons were performed using the Mann–Whitney U test and are reported as Z statistics. Age-adjusted comparisons were performed using rank-based regression models in which continuous outcomes were rank-transformed and age was included as a covariate. *p* values below 0.001 are reported as <0.001.

### 3.2 Correlation analysis

**Figure 6:**
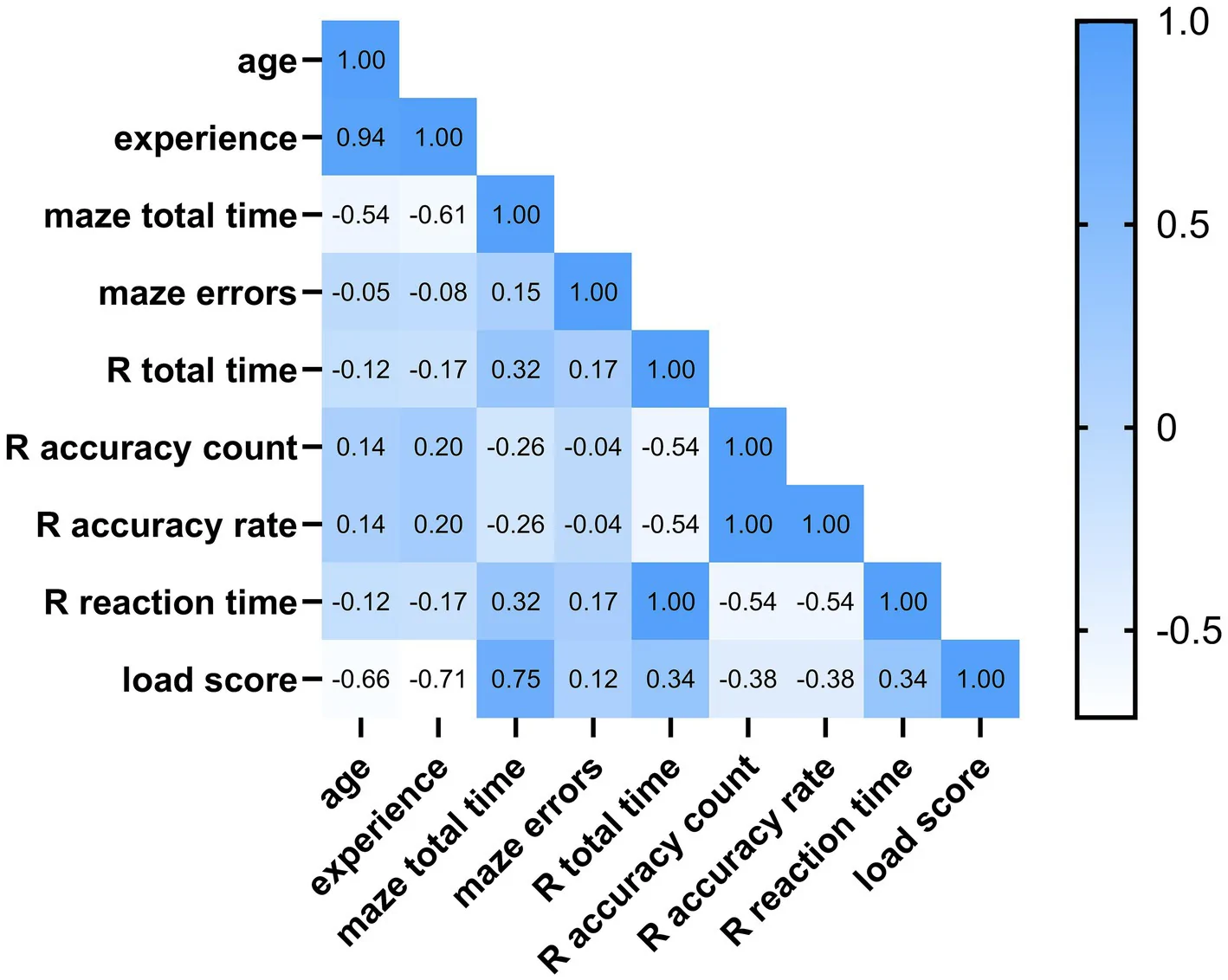
Correlation heatmap for age, flight experience, spatial task performance, and subjective cognitive load.

Correlation analyses revealed significant associations among demographic, performance, and subjective measures. Age was strongly positively correlated with total flight experience (r = 0.94, *p* < 0.001), indicating that greater age was associated with more flight hours in this sample. Greater flight experience was associated with shorter maze completion time (r = −0.61, *p* < 0.001) and lower cognitive load (r = −0.71, *p* < 0.001). Subjective cognitive load was positively correlated with maze completion time (r = 0.75, *p* < 0.001) and negatively correlated with mental-rotation accuracy (r = −0.46, *p* < 0.001), indicating that higher perceived load was associated with poorer task performance (Figure 6).

### 3.3 Regression analysis

A hierarchical multiple linear regression was conducted with maze total time as the dependent variable. Age was entered in the first block, followed by cognitive load, group, and flight experience in the second block. Model 1, containing only age, explained 24.1% of the variance in maze total time (R^2^ = 0.241, *F* (1, 158) = 50.207, *p* < 0.001). The inclusion of cognitive load, group, and flight experience in Model 2 increased the explained variance (Delta R^2^ = 0.341, *p* < 0.001), with the full model accounting for 58.2% of the variance (R^2^ = 0.582, *F* (4, 155) = 53.904, *p* < 0.001). In the final model, cognitive load was the only significant predictor (beta = 0.732, t = 10.069, *p* < 0.001) (Table 1). Age, flight experience, and group were not significant after cognitive load was included.

A second hierarchical multiple linear regression examined factors influencing R-letter accuracy count. Model 1, containing only age, explained 3.4% of the variance (R^2^ = 0.034, *F* (1, 158) = 5.478, *p* = 0.021). Model 2 explained 10.0% of the variance (R^2^ = 0.100, *F* (4, 155) = 4.309, *p* = 0.002). In the final model, cognitive load was the only significant predictor (beta = −0.308, *p* = 0.004), indicating that higher load was associated with fewer correct responses (Table 2). Age, flight experience, and group were not significant in the final model.

### 3.4 Exploratory statistical mediation analysis

Mediation analysis was conducted using PROCESS Model 4 to examine whether cognitive load statistically accounted for the association between pilot status and maze task performance, controlling for age. The bias-corrected bootstrap method with 5,000 samples was used to assess the indirect effect.

In the full sample, the total effect of group on maze time was significant: the student group took longer to complete the maze task than the pilot group (c = 33.82, SE = 9.20, *p* < 0.001). After cognitive load was included as a mediator, the direct effect was not significant (c’ = −1.28, SE = 7.94, *p* = 0.873), whereas the indirect effect was significant (ab = 35.10, Boot SE = 8.36, 95% Boot CI [20.51, 53.67]). The student group reported higher subjective cognitive load (path a = 8.63, *p* < 0.001), and higher cognitive load was associated with longer task time (path b = 4.07, *p* < 0.001). Because the indirect effect exceeded the total effect, this pattern may reflect inconsistent mediation or suppression and should not be interpreted as complete mediation. Consistent with recommendations for cross-sectional mediation (Maxwell and Cole, 2007), these results should be interpreted strictly as exploratory statistical mediation. Since cognitive load and performance were measured concurrently at a single time point, and the indirect effect exceeded the total effect (suggesting inconsistent mediation or suppression), we cannot infer causal mechanisms or temporal precedence. Future longitudinal studies are required to confirm these associative patterns (Table 4).

**Table 4:** Mediation analysis of the effect of pilot status on maze task performance through cognitive load in the full sample (*N* = 160).

Path/effect	B (SE)	beta	t	*p*	95% CI lower	95% CI upper
Total effect: Group → Maze time	33.820 (9.200)	0.366	3.676	<0.001	15.65	51.99
Direct effect: Group → Maze time	−1.275 (7.936)	−0.014	−0.161	0.873	−16.95	14.40
Cognitive load → Maze time	4.067 (0.399)	0.613	10.193	<0.001	3.28	4.85
Group → Cognitive load (a)	8.630 (1.431)	0.600	6.033	<0.001	5.80	11.46
Mediation effect (a x b)	35.095 (8.355)	0.370	—	—	20.51	53.67

Group coding: 0 = pilot, 1 = student pilot. Age was included as a covariate. The confidence interval for the indirect effect is a bias-corrected bootstrap 95% CI based on 5,000 samples. R^2^ for the model of maze time = 0.581.

Exploratory analyses in the pilot subgroup (*n* = 80) showed that flight experience had no significant total effect on maze task duration (c = −0.0119, *p* = 0.120), and the indirect effect through cognitive load was not significant (ab = −0.0066, 95% Boot CI [−0.0182, 0.0004]). Cognitive load remained a significant predictor of maze performance (b = 3.0415, *p* < 0.001), whereas flight experience did not significantly predict cognitive load (a = −0.0022, *p* = 0.110) (Table 5).

**Table 5:** Mediation analysis of the effect of flight experience on maze task performance through cognitive load in the pilot subgroup (*n* = 80).

Path/effect	B (SE)	beta	t	*p*	95% CI lower	95% CI upper
Total effect: Flight experience → Maze time	−0.0119 (0.0076)	−0.157	−1.574	0.120	−0.027	0.003
Direct effect: Flight experience → Maze time	−0.0053 (0.0065)	−0.070	−0.812	0.420	−0.018	0.008
Cognitive load → Maze time	3.0415 (0.5413)	0.603	5.619	<0.001	1.96	4.12
Flight experience → Cognitive load (a)	−0.0022 (0.0014)	−0.175	−1.616	0.110	−0.005	0.001
Mediation effect (a x b)	−0.0066 (0.0044)	−0.106	—	—	−0.0182	0.0004

X = flight experience in hours. Age was included as a covariate. The confidence interval for the indirect effect is a bias-corrected bootstrap 95% CI based on 5,000 samples and includes zero, indicating a non-significant indirect effect. R^2^ for the model of maze time = 0.374.

## 4 Discussion

This study examined spatial navigation, mental rotation, and subjective cognitive load in pilots and student pilots using VR-based spatial cognition tasks. While previous studies have examined spatial ability in pilots using VR (Zhang et al., 2023) or investigated cognitive load in flight simulators, few have integrated these streams of research to quantitatively assess whether cognitive load mediates group differences in ecologically enriched VR environments. Moreover, the specific contribution of accumulated flight hours versus the qualitative shift from trainee to licensed pilot remains underexplored. This study aims to fill this gap by simultaneously assessing navigation and object-rotation abilities, quantifying subjective cognitive load, and employing mediation analysis to explore the statistical pathways linking pilot status to spatial performance.

The main findings were that pilots completed the VR maze faster, achieved better performance on selected R-letter rotation indicators, and reported lower subjective cognitive load than student pilots (Hart and Staveland, 1988). Subjective cognitive load was closely associated with maze completion time and R-letter accuracy, and cross-sectional mediation analysis suggested a significant indirect association between pilot status and maze performance through cognitive load. However, because pilots were older and had substantially greater flight experience than student pilots, these differences should be interpreted strictly as pilot-status-associated differences. Due to the cross-sectional design and the high collinearity between age and flight hours, we cannot infer definitive causal effects of flight experience (Nori et al., 2021).

The maze task requires participants to integrate environmental cues, update their position, maintain route information, and execute goal-directed navigation. These processes overlap with spatial orientation demands in aviation, including maintaining heading, updating self-position, and integrating visual–spatial information during movement. The finding that pilots completed the VR maze faster than student pilots is consistent with previous evidence that aviation expertise is associated with more efficient spatial processing and simulator performance (Causse et al., 2011; Keller and Sutton, 2018). VR-based navigation tasks may provide a more ecologically enriched assessment of spatial orientation than conventional two-dimensional tests because they allow active movement, dynamic cue integration, and real-time interaction (Coutrot et al., 2019). Nevertheless, the VR maze remains an analogue spatial navigation task rather than a direct simulation of flight operations, and the results should therefore be generalized cautiously.

The R-letter mental rotation task differs from the maze task because it primarily requires object-based spatial transformation rather than route integration or self-location updating. Classic mental rotation studies have shown that response time and accuracy vary with angular disparity and the mental transformation demands of the stimulus (Shepard and Metzler, 1971). Therefore, pilots’ advantage in the R-letter task may reflect more efficient spatial transformation or response strategies, but this task may be less directly related to aviation navigation than the VR maze. The attenuation of some group differences after age adjustment suggests that age-related cognitive development, selection effects, or baseline spatial ability was statistically associated with the observed differences. In addition, the high accuracy in both groups indicates a possible ceiling effect, which may have reduced the sensitivity of the R-letter task to distinguish experienced pilots from student pilots.

This study discovers significant differences in spatial cognition levels between pilots and flight trainees, with age differences being a contributing factor (Tsang and Shaner, 1998). Experienced pilots demonstrated better performance on selected indicators in both tasks, consistent with previous findings of expertise-associated differences in cognitive performance. These findings are consistent with the possibility that professional aviation training and experience are associated with more efficient cognitive resource allocation (Bastiaens et al., 2017). In flight mission scenarios, it is plausible that prolonged training and practical experience are associated with more automated processing of spatial information. While van Merrienboer and colleagues demonstrated in experimental settings that deliberate practice can accelerate automation enabling highly experienced individuals to perform tasks with reduced cognitive load (Frèrejean et al., 2025).

Subjective cognitive load was strongly associated with maze completion time and R-letter accuracy. Regression analyses further indicated that cognitive load was the strongest associated factor of maze time and the only significant predictor of R-letter accuracy in the final model. These findings suggest that subjective cognitive load may help explain variability in spatial task performance in VR environments. This pattern suggests that experienced pilots may employ different cognitive strategies during spatial tasks, which may be reflected in differences in perceived cognitive demand. However, whether these differences result from training, selection effects, or pre-existing characteristics remains unclear. These findings support the theory of skill transfer and expert performance, which posits that domain-specific experience optimizes strategies for allocating cognitive resources. The pilots’ advantages may partly stem from an experience-modulated pattern of more efficient cognitive resource utilization (Kalyuga et al., 2003). The correlation between the total duration of the maze and spatial cognitive load highlights the close relationship between task time and cognitive load, supporting the theoretical assumption that “time cost is a core dimension of cognitive load” (Barrouillet et al., 2004). In other words, the advantages observed in experienced pilots likely stem not from static “superior abilities” unaffected by task difficulty, (Adams and Ericsson, 2000) but rather from a dynamic capacity to maintain low cognitive load under challenging conditions—a trait consistent with the “adaptive expert” theory of professional skill development, where expert proficiency arises from optimized cognitive resource allocation and load management strategies rather than mere enhanced capability.

The mediation analysis showed a significant indirect association between group status and maze performance through subjective cognitive load. However, because the indirect effect exceeded the total effect and the study was cross-sectional, the results should not be interpreted as evidence of complete mediation or a causal mechanism. Rather, lower subjective cognitive load may partly account for the observed group differences in VR maze performance. However, because this study used a cross-sectional design and because flight hours were highly correlated with age, the results cannot establish that flight experience causally reduced cognitive load or improved spatial performance. The performance advantage observed in pilots was statistically associated with lower subjective cognitive load during task execution, rather than merely reflecting a static ‘pilot’ identity they experienced during task execution. This finding positions the advantage of professional skills as stemming from optimized cognitive processing efficiency rather than static ability differences, strongly supporting the application of cognitive load theory in explaining complex real-world skills (Adams and Ericsson, 2000).

Exploratory analyses within the pilot subgroup showed no significant linear association between total flight hours and maze performance or cognitive load. This finding suggests that, within qualified pilots, additional flight hours may not translate linearly into improved performance on standardized VR tasks. Alternatively, the task may have been insufficiently difficult to detect differences among pilots, or important variables such as aircraft type, recent flight activity, simulator exposure, and training quality may be more informative than total flight hours alone.

Collectively, these findings are consistent with a pattern characterized by a marked improvement in cognitive efficiency upon transition from trainee to qualified pilot. This is manifested as a significant reduction in cognitive load; subsequently, within-group differences suggest that the linear accumulation of experience may enter a plateau phase, with its benefits potentially manifesting in more complex, higher-order, or dimensions of ability that are more closely related to “experience quality”. VR tasks that are sensitive to subjective cognitive load may be useful for aviation-training research and assessment development; physiological indicators including eye tracking, electroencephalography, and functional neuroimaging can be adopted in future research to further elucidate the neural and attentional mechanisms underlying spatial cognition and cognitive load within aviation scenarios.

## Data Availability

The raw data supporting the conclusions of this article will be made available by the authors, without undue reservation.
