# Supplementary material for: Differences in spatial cognitive performance and cognitive load between pilots and student pilots in virtual reality environments
Source: Front Psychol. 2026 Aug 12;17:1903114. doi: 10.3389/fpsyg.2026.1903114 (PMC13506879; doi:10.3389/fpsyg.2026.1903114)
Supplement: Supplementary file 1 [file Data_Sheet_1.DOCX]

Questionnaire Instructions

1. Main Tools Used in This Study The self-designed questionnaire evaluates cognitive load during spatial cognition testing. Specifically developed for VR environments, it incorporates theoretical frameworks including Wickens 'Multi-Resource Theory, Brooks' Spatial Cognition Model, and the NASA-TLX scale (revised version), comprising 18 items across 6 dimensions. Preliminary experiments using 230 questionnaires demonstrated a Cronbach's Alpha coefficient of 0.825, indicating strong internal consistency.

Adapted NASA-TLX-Based VR Spatial Cognition Task Load Questionnaire

Name: Gender: Male (), Female (); Age:

Contact Information: The VR Spatial Cognition Task Load Questionnaire comprises 18 items. Designed to objectively assess your actual experience during task completion, all responses will be anonymized. Please provide objective evaluations based on your actual test experience, responding quickly according to your initial impression. Under each item in the table, select the closest answer from the five options listed and mark it with a "√". The evaluation results will be used solely for research purposes and will be kept strictly confidential. We appreciate your professional and objective participation in this assessment. Thank you for your cooperation!

1. Does the task require me to quickly analyze the question and make a decision?

A. Not at all necessary B. Not necessary C. Neutral D. Necessary E. Very necessary

2. When facing complex issues requiring analysis, does my decision-making speed noticeably slow down?

A. Not at all B. Not very well C. Generally D. Relatively slow E. Very slow

3. Can unexpected situations in the task help me stay calm? (Reverse scoring)

A. Very tense B. Relatively tense C. Generally D.Relatively easy E. Very easy

4. Do I need to frequently turn my head to navigate the maze?

A. Not at all necessary B. Not necessary C. Generally D. Necessary E. Very necessary

5. How well can I align virtual object positions with real-world hand movements?

A. Perfectly achievable B. Achievable C. Generally D. Not very achievable E.

Completely unachievable

6. Does rapid head rotation cause visual blurring or disorientation?

A. Not at all B. Not very much C. Generally D. Relatively noticeable E. Very noticeable

7. Do you ever feel time is short during time-limited tasks?

A. Not at all B. Not very much C. Generally D. Not enough E. Very insufficient

8. Are you forced to speed up your operations due to time constraints or external pressure?

A. Not at all B. Not very much C. Generally D. Relatively noticeable E. Very noticeable

9. Even under time pressure, I will carefully complete all steps and thoroughly consider them.

A. Carefully consider and complete B. Consider and complete C. Generally D. Not very much consider E. Completely disregard and skip randomly

10. Does understanding graphic rules require mental effort?

A. Very easy B. Relatively easy C. Average D. Relatively difficult E. Very difficult

11. Does it require repeated attempts to achieve precise steering?

A. Not at all B. Not required C. Generally D. Required E. Very required

12. When faced with unexpected obstacles or challenges, do I need to engage in careful reflection and analysis?

A. Not at all B. Not very well C. Generally D. Will try my best E. Very hard

13. How significant is the deviation between the operational actions and the expected outcomes?

A. Completely no deviation B. Minimal deviation C. Average D. Significant deviation E. Extremely significant deviation

14.I find it hard to precisely control the movement trajectory of virtual characters or tools.

A. Not difficult at all B. Not too difficult C. Generally D. Relatively difficult E. Very difficult

15.There is a noticeable delay between my motor actions and the visual feedback.

A. No delay at all B. Minimal delay C. Average D. Relatively noticeable delay E. Significant delay

16. Do you feel frustrated after making multiple mistakes?

A. Not at all B. Not very C. Generally D. Can be quite frustrating E. Very frustrating

17. Does operational error cause frustration?

A. Not at all B. Not very much C. Generally D. Relatively strong E. Very strong

18. Could a single operational error make me increasingly anxious when tackling subsequent tasks?

A. Not at all B. Not very much C. Generally D. Relatively obvious E. Very obvious

2Brief description of the data collection procedure: Method: On-site distribution of paper questionnaires. Process: Participants sign the informed consent form-> Complete the spatial cognition experiment-> Complete Questionnaire B-> On-site collection/submission.

Quality control: All investigators underwent standardized training; reverse scoring questions were implemented; and data were double-entered for verification.
